# Loneliness among Middle-Aged and Older Middle-Aged Adults in Russia (Saint Petersburg) before and during COVID-19 Pandemic

**DOI:** 10.3390/ijerph18189889

**Published:** 2021-09-20

**Authors:** Olga Strizhitskaya, Marina Petrash, Inna Murtazina, Gayane Vartanyan, Anton Shchukin

**Affiliations:** Faculty of Psychology, Saint Petersburg State University, 199034 Saint Petersburg, Russia; m.petrash@spbu.ru (M.P.); i.r.myrtazina@spbu.ru (I.M.); g.vartanyan@spbu.ru (G.V.); a.v.schukin@spbu.ru (A.S.)

**Keywords:** loneliness, solitude, adulthood, COVID-19, positive loneliness, emotional loneliness, isolation

## Abstract

Loneliness has been considered a major challenge since long before the pandemic. Changes caused by the COVID-19 pandemic included modifications in social communications and activities. Thus, it was expected that loneliness would increase during the pandemic. The first studies of loneliness during the pandemic revealed inconsistent results. We hypothesized that physical isolation led to changes in the quality of relationships; thus, loneliness trends could be different from those predicted. For our study we used methods to measure loneliness: the Social and Emotional Loneliness Scale (SELSA-S) for Adults and Older Adults; the Multidimensional Inventory of Loneliness Experience; and demographic data. Participants were middle-aged and older middle-aged adults (*n* = 457) aged 35–59 (M = 45.5, SD = 6.88, 35.4% males). Participants came from two studies: Study 1 consisted of 280 participants aged 35–59 (M = 44.8; SD = 6.93; 29.6% males), the study was conducted before the pandemic in late 2019; participants in Study 2 were adults (*n* = 177) aged 35–59 (M = 46.5; SD = 6.68; 44.6% males), data were collected in the fall of 2020. The results did not confirm increase in loneliness; moreover, participants reported lower scores of loneliness in some domains. Regression analyses showed that general experience of loneliness was predicted by different loneliness characteristics in pre-pandemic and pandemic age groups. We found some similar mechanisms that were activated within different situations. Our results confirmed the complex nature of loneliness, they argue that pandemic effects were not limited to increase in loneliness and that the mechanism of loneliness can adjust to environmental factors.

## 1. Introduction

Loneliness is conceptualized as a subjective discrepancy between the actual and the desired social relationships in terms of closeness, intimacy, emotional support and connectedness [1]. Loneliness is considered to be a painful experience and a predictor of a variety of heath conditions, including risk for early mortality [2,3]. At the same time, Capiocco [4] argues that while the negative effects of loneliness are considerable, temporary periods of loneliness may also have some positive effects and serve for self-reflection and self-analysis. Capiocco and his colleagues suggest that loneliness might be an evolutionary mechanism. They point out that the degree to which one is sensitive to pain caused by isolation and lack of communication may vary: those less sensitive to pain might be more prone to discoveries and seek new opportunities, while those more sensitive might be more cautious and protective. Although it is only one way of interpreting loneliness, it gives a particular perspective on the possible variability of loneliness mechanisms.

Another concept related to loneliness and lack of communication is solitude [5]. While loneliness has more widespread negative connotations, solitude describes physical absence of partners for communication and might be both negative and positive. Particularly, people may vary in their experiences of solitude depending on cultural and situational context [6]. For instance, in the study by Lay et al. [6], people from individualistic countries were expected to benefit from solitude more, as it was consistent with the basic values of freedom and independence, while in collectivistic countries it might lead to social maladjustment. Contemporary research approaches loneliness from unidimensional and multidimensional perspectives [7]: the former assumes that loneliness is a generalized experience while the latter acknowledges the possibility of existence of different components or facets of loneliness.

Researchers underline that loneliness is reported across all ages and periods of adulthood [8]. On the basis of a meta-analysis of 78 longitudinal studies, they argued that variability of loneliness to some extent was explained by personal experience and thus was not directly associated with age. The pandemic situation may be considered to be a personal situation, since the way one processes this situation is determined by both objective social situations, including real restrictions and personal reactions, attitudes, coping strategies, etc., defined by one’s psychological characteristics.

On 11 March 2020, the World Health Organization announced the global pandemic caused by an outbreak and transmission of SARS-CoV-2 (COVID-19), which resulted in application of a variety of restrictions in most countries all over the globe [9]. One of the main means for decreasing the spread of the COVID-19 was the physical isolation of people: lockdowns, social distancing, and restrictions on social networking and activities. This induced isolation was expected to affect the experiences of loneliness [10,11,12]. In the Russian Federation, lockdown was announced on 30 March 2020 and lasted until 11 May 2020. During that period people had to stay at home, except for shopping for necessary groceries and workers whose jobs were crucial for survival systems (like maintaining electricity, water, etc.). Security measures included social distancing, wearing masks and gloves, and using sanitizer whenever outside one’s apartment. Travel outside the country and between regions inside the country was closed. From 11 May 2020 the restrictions were softened, and people were given more freedom to go outside their apartments, but public places like restaurants and gyms remained closed, and travel remained restricted. By September 2020, the restrictions were limited to social distancing, wearing masks and gloves, using sanitizer, and limitations on public meetings. Travel outside the country was still limited, but restrictions for travel inside the country were cancelled [13].

Since the start of the pandemic, a solid body of research has addressed different aspects and effects of COVID-19, including loneliness. Nevertheless, the results still leave room for discussion [14]. The studies that have addressed loneliness in times of COVID-19 differ in terms of their design, the methods used, longitudinal or cross-sectional data, and unidimensional or multidimensional approaches to loneliness.

A significant increase in loneliness was predicted and reported in studies conducted in the U.S. on cross-sectional samples [11,15]. At the same time, a study by Tull and colleagues [16] showed that COVID-19-related impact, based on self-reports, negatively predicted loneliness. One possible explanation for this was suggested by Sutin and colleagues [17]: they proposed that the pandemic caused not only unpredictable distress and limitations, it also led people to provide more support. Thus, despite some social restrictions, the quality of communications could improve.

Increase in loneliness was revealed in a study from the UK comparing two population-based studies: the UK Household Longitudinal Study and the COVID-19 Social Study [18]. Although the authors confirmed an increase in loneliness during COVID-19, they also argued that the risk factors remained the same as before the pandemic, and vulnerable groups were more likely to experience significantly increased levels of loneliness compared to low-risk groups.

Contrary to the studies cited above, a study conducted by Luchetti et al. [19] on a sample of adults aged 18–98 showed no significant effects of the pandemic on experiences of loneliness; moreover, their results revealed an increase in social support despite physical isolation and use of alternative methods of communication. A population-based study conducted in Germany [20] also did not confirm increases in loneliness; while older adults were expected to show significant changes in loneliness, the most vulnerable group appeared to be young adults (under the age of 30). Similar results were presented in the UK by Groarke et al. [21]: the loneliness rates, revealed in the cross-sectional study of adults over 18 years old, were moderately high (27%); nevertheless, these rates were similar to before-pandemic rates, and none of the factors related to COVID-19 predicted loneliness.

Interesting results were reported by Stieger, Lewetz and Swami [22], who used systematic observation for assessment of well-being and loneliness in an Austrian sample. Similar to the study by Groarke et al., Stieger et al. reported that pre-pandemic factors such as being outdoors vs. being indoors predicted better well-being and lower loneliness. At the same time, their systematic observation showed that loneliness had a non-linear trend and depended on lifestyle factors.

Multidimensional studies have reported a more complicated picture for loneliness. Results from a Dutch population-based longitudinal study [23] showed that while emotional loneliness increased during the pandemic, social and social emotional loneliness did not show any significant changes. There have also been studies claiming that loneliness rates due to lockdowns caused by COVID-19 were high or higher (often conducted during or right after the first lockdown), for example [24,25,26,27,28], but data were not compared with pre-pandemic rates. A rapid review, reported by Pai and Vella [14], outlined the inconsistencies in the existing studies: differences in samples in terms of demographic characteristics, and discrepancies in the results.

In summary, the data related to COVID-19 loneliness do not suggest clear trends or associations. Longitudinal studies tend to report no or little differences in the mean levels of loneliness. They confirm that pre-pandemic and pandemic factors, predicting loneliness, remain the same; and do not support the ideas of a significant role of the pandemic in increasing levels of loneliness. Cross-sectional studies were more likely to report high absolute means of loneliness during the pandemic, particularly within lockdown periods. From a methodological perspective, most studies used a unidimensional approach (UCLA loneliness scale based on three self-report items, each item to be estimated on a scale from 1 (hardly ever) to 3 (often), all items are summed to give a total score) that might not be sensitive to particular facets of loneliness. Finally, from a lifespan developmental perspective, few studies concentrated on effects for particular age groups, such as middle-aged or older middle-aged adults, generalizing to the adult population. To date, there have not been any studies that have addressed changes in solitude during the COVID-19 pandemic.

Our study aimed to approach loneliness using a multidimensional model. We focused on comparative analysis of pre-pandemic and pandemic experiences of loneliness in two demographically similar samples, living in similar social and economic conditions, evaluated with the same instruments that addressed loneliness and the positive aspect of solitude. We hypothesized that general scores of loneliness in pre-pandemic and pandemic samples would be comparatively similar. At the same time, we expected that some facets of loneliness such as family emotional loneliness would decrease due to more time, concerns and support that people were to some extent induced to provide by the pandemic situation. We also expected a decrease in positive aspects of solitude scores because of two assumptions. First, induced isolation limited the opportunities of voluntarily solitude (since many middle-aged adults lived with their nuclear families or partners). Second, similar to the ideas suggested by Sutin [15], the pandemic put most people in similar conditions of struggling against COVID-19 that could temporarily shift the focus from the needs of the self to those of others.

## 2. Materials and Methods

### 2.1. Data Collection and Research Design

This study started as a bi-cultural project on loneliness “Loneliness vs. independence across lifespan: perspectives and insights from Bulgaria and Russia” and aimed at complex analysis of loneliness in different contexts. The study was conducted in Saint Petersburg, Russia before and during the COVID-19 pandemic: Study 1 took place from November 2019 to February 2020 while there were no restrictions and the pandemic had not yet been globally recognized; Study 2 was conducted from October to December 2020, when the pandemic restrictions were moderate.

The period between 20 and 65 years is often referred to as adulthood (for example, APA [29]). However, the period of 45 years is too large to be treated as homogeneous in terms of psychological or social characteristics. Therefore, in lifespan developmental studies, adulthood is usually split into sub-periods: early adulthood (approximately 20–30/35 years), middle adulthood (approximately 35–45 years), and older middle adulthood (approximately 45–60 years). These periods differ in terms of developmental tasks to be solved. In our study, we focused on the middle and older periods of adulthood. Developmental tasks for the middle period of adulthood include career development and family-related goals, including raising young or teenage children. Developmental tasks for the upper end of adulthood include the peak of career achievements and related goals, and probably the main task is related to getting ready for retirement and aging [30].

In our study, participants were middle-aged and older middle-aged adults (*n* = 464) aged 35–59 (M = 45.5, SD = 6.88, 35.4% males). Seven participants were excluded from the study (3 from Study 1 and 4 from Study 2) because their questionnaires were incomplete. Participants of Study 1 were adults (*n* = 280) aged 35–59 (M = 44.8; SD = 6.93; 29.6% males), 74% had university degree, 58% were married, 10.2% reported living with a partner, 16% divorced, 14% single. Participants of Study 2 were adults (*n* = 177) aged 35–59 (M = 46.5; SD = 6.68; 44.6% males), 81.5% had university degree, 61.8% were married, 10.1% reported living with a partner, 16.3% divorced, 10.1% single. Participants came from different professional backgrounds: education, medicine, management, accounting, engineering etc. They had similar income and reported their income as “have enough money for everyday life” (51%) and “have enough money for everyday life and can afford vocation travel” (47%). In the pandemic group there were no participants involved in services or jobs directly related to COVID-19 (such as doctors, for example) or working in the services related to city functioning (like gas and electricity companies, for example). All the participants were employed.

For analysis purposes, participants in each study were divided into two age groups: 35–44 and 45–59. Demographic data are presented in Table 1.

Participants were recruited via community (information was given at social meetings) and social networks (like Facebook and Vkontakte). There was a preliminary talk where participants were explained the aims of the study, its focus, main procedures and their rights. The talk was performed in person or via conference means of communication. The talk was individual. No incentives were given to the participants. Participation in the study was anonymous, and no personal data such as names, phone numbers or addresses were collected. The questionnaires were distributed in person and via online forms (google forms). The links to google forms were sent only to participants who had completed the preliminary talk and given their consent to participate in the study. The research design, procedures, measures and sampling were approved by the review board of the Russian Foundation for Basic Research (project No. 19-513-18015). Informed consent was obtained from all the participants and the Helsinki declaration was respected.

Our research questions were:

(1) did the pandemic situation affect experiences of loneliness among participants in our sample?
(2) were there age effects in pandemic groups?
(3) was the general experience of loneliness variance explained by the same predictors in all defined groups?

We hypothesized that:

- Based on longitudinal studies [19,20,21] and ideas suggested by Sutin [17], we expected no or small effects of the pandemic on the general scores on loneliness. We also hypothesized that partial scores of loneliness, related to particular social background might decrease because of induced increase in objective time people spent with their families;
- Based on socioemotional theory [31], which argues that motives for social interaction change across the lifespan, we hypothesized that these changes might affect experiences of loneliness in different adult age groups;
- Based on the same theory [31], we assumed that generalized experience of loneliness in different age groups coming from pre-pandemic and pandemic samples could be explained by different partial characteristics of loneliness.

### 2.2. Measures

Our studies were concentrated on the idea that loneliness is a complex phenomenon that requires a multifaceted measurement. For our studies we used two questionnaires that assess loneliness in different spheres and the quality of loneliness experiences:

(1) The Social and Emotional Loneliness Scale (SELSA-S) for Adults and Older Adults [32]. The Scale consists of 19 items that are scored from 1 to 5. The Scale includes four subscales: family emotional loneliness, non-family emotional loneliness, loneliness in romantic relationships, and romantic emotional loneliness,

(2) The Multidimensional Inventory of Loneliness Experience [33]. The Inventory has 24 items scored from 1 to 4. The Inventory consists of three subscales: general experience of loneliness, dependence on communication, and positive loneliness.

Our measures were:

*Family emotional loneliness* [32] had 7 items and described loneliness related to family interaction.

*Non-family emotional loneliness* [32] consisted of 6 items and addressed loneliness, experienced in non-family background, primarily in relationships with friends.

*Loneliness in romantic relationships* [32] consisted of 3 items and focused on the presence or absence of the romantic relationships. This scale described physical absence of romantic relationships.

*Romantic emotional loneliness* [32] was based on 3 items and addressed loneliness in existing romantic relationships. This measure expressed experiences when one had a romantic partner but was not satisfied with the emotional feedback from him/her.

*General experience of loneliness* [33] had 8 items that reflected general undifferentiated experience or feeling of loneliness.

*Dependence on communication* [33] included 8 items and addressed negative attitudes to the very idea of being alone, seeking for communication and relationships at whatever cost not to be alone.

*Positive loneliness* [33] consisted of 8 items and reflected attitudes to loneliness as a resource for self-understanding and self-development. This measure included the ability to value the moments of loneliness and to intentionally create such moments.

### 2.3. Statistical Analysis

Data were processed using the statistical program Statistical Package for Social Sciences SPSS (v20.0, SPSS Inc., Chicago, IL, USA). We used *t*-test and MANOVA for between-group effects, linear and non-linear regression to test possible age effects with age as a continuous variable, and regression analysis using the Enter method for loneliness characteristics. For all methods, we used Bootstrap procedures to confirm the significance of the results.

## 3. Results

To address Research Question 1, we tested our data for significant effects of pandemic period (pre-pandemic and pandemic group) using *t*-test for independent samples (Table 2). The results showed that general experience of loneliness, positive loneliness, family emotional loneliness and loneliness in romantic relationships were lower in the pandemic group. Dependence on communication, non-family emotional relationships and romantic emotional relationships were similar in both pandemic groups. The significance of the differences was confirmed by the bootstrapping procedures. Thus, our results did not confirm an increase in loneliness during the pandemic; moreover, participants reported lower scores of loneliness in some domains during the pandemic than in the pre-pandemic period.

To approach Research Question 2, we used multivariate ANOVA (MANOVA) for independent factors “Pandemic group” (0; 1), “Age” (1; 2) and “pandemic period × age”. MANOVA did not reveal any significant effect for age or pandemic period × age. Means and standard deviations for all age groups are presented in Table 2.

The lifespan development of an adult is a heterogeneous process that does not have definite markers that would clearly state transition from one age period to another. Thus, we concluded that age effects for loneliness could be predicted by non-linear regression models. Comparative analysis of linear, quadratic and cubic regression models for characteristics of loneliness as the dependent variables and age as an independent variable presented. We conducted regression analysis for loneliness variables that showed significant statistical differences between pre-pandemic and pandemic periods. The results revealed that neither linear nor non-linear models fitted the empirical data. This result suggests that multifaceted characteristics of loneliness may vary across the adult lifespan, but that this variation cannot be explained by age effects.

To address Research Question 3, we conducted regression analysis in the pre-pandemic and pandemic samples separately for age groups 35–44 and 45–59 (Table 3 and Table 4). The dependent variable was *General experience of loneliness*, as it was the most undifferentiated and generalized variable of loneliness we used; the rest of the characteristics of loneliness were included in the regression model as independent variables. The Enter method was used, and the significance of the results was controlled by bootstrapping procedures.

In the pre-pandemic sample aged 35–44, general experience of loneliness was predicted by all characteristics of loneliness except loneliness in romantic relationships. This model explained 62.1% of the general experience of loneliness variance (R^2^ = 0.621). These data showed that before the pandemic, middle-aged adults had a complex structure of loneliness characteristics, and their experience of loneliness was based on multifaceted relations and interactions that affected the overall experience of loneliness.

Older middle-aged adults showed a slightly different picture. General experience of loneliness was associated only with emotional loneliness within different social situations—family interaction, non-family interaction, romantic interaction. The model accounted for 61.1% of general experience of loneliness variance. Thus, we can assume that closeness and connectedness were more important than the need for whatever communication or need for “personal” space.

Analysis of general experience of loneliness in the pandemic sample showed drastically different results. For middle-aged adults, general experience of loneliness was predicted only by the variable non-family emotional relationships, which accounted for 52.3% of the dependent variable variance. We suggest, that during the pandemic period, induced isolation led to a closer interaction among family and romantic partners, since 68.2% were married or lived with their partner. Before the pandemic, adults were leaving their homes to go to work on a daily basis (distant work was not that widespread among the people from 35 to 59). During the pandemic for several months (from late March to early July) people were physically staying at home due to state restrictions. Thus, we suggest that the variance of general experience of loneliness could be caused by the type of relationships outside the family; by reduced interactions with friends and colleagues.

For older middle-aged adults, the strongest predictors of general experience of loneliness were family and non-family emotional relationships. Nevertheless, dependence on communication and positive loneliness also had predictive power. Interestingly, though dependence on communication and positive loneliness address different, almost opposite variants of interactional patterns, in this model they both positively predicted loneliness. Together, dependence on communication, positive loneliness, family and non-family emotional loneliness explained 56.3% of general experience of loneliness variance. These results suggest that older middle-aged adults from the pandemic sample might experience contradictory tendencies based on the emotional feedback they receive from their close social contacts.

## 4. Discussion

Our results support the longitudinal studies [19,20,21] that argue that means scores of loneliness were maintained throughout the pandemic. Contrary to expectations [11], we found that some characteristics of loneliness were reported at lower rates during the pandemic than during the pre-pandemic sample. Our data illustrate that the structural specifics of loneliness may be sensitive to pandemic effects. While some studies [18] reported that the factors of vulnerability to loneliness before and during pandemic were similar, our results demonstrated several distinctive mechanisms. We assumed that such differences could be attributed to the processes of psychological adjustment to isolation from the general public, a forced increase in communication with close social networks and the increased ambiguity of social situations in general.

One of the interesting results we found in our study was that dependence on communication, emotional loneliness and positive loneliness predicted general experience of loneliness with the same directionality. That is, when one experienced a need for communication, he/she was also voluntarily seeking solitude and experiencing emotional loneliness. Another scenario for this model is that those who received support and connectedness from close social contacts were not looking for solitude and were not dependent on communication. This result could illustrate the argument that when one lives in an unfavorable situation—for example, with a lack of understanding in one’s family—he or she needs such voluntary solitude to find a way to resolve this situation. At the same time, lack of mutual understanding would lead to a search for relations in which one could feel affiliation, support and connectedness. We could elaborate this idea and speculate that dependence on communication (or seeking of and need for communication) and positive loneliness (or voluntary solitude) were not opposite characteristics. They might be two different strategies to resolve the same issue: seeking social support (dependence on communication) and distancing (positive loneliness or solitude). Consequently, in particularly difficult situations, one could activate both strategies to resolve the situation. We found this mechanism in two groups: pre-pandemic middle-aged adults and pandemic older middle-aged adults. We could speculate that in the first group, this mechanism could be activated because of high overload with multifaceted tasks. For example, a person who is overwhelmed with daily tasks and challenges, and does not receive emotional support from relatives and friends, would at the same time want more communication but prefer to stay alone if the actual social background cannot satisfy the emotional needs. The second group was in the ambiguous situation of the pandemic. The society and people were shocked with the fact that relatively young people, aged 50–60, were dying from a new unknown disease within quite short periods of time, given that the life expectancy in Russia is 79.85 years for women and 71.34 for men [34]. That would lead to a new value of existing relationships; we could argue that in such a situation people would need more emotional support and would use more than one strategy to gain it.

We found that non-family emotional loneliness was a predictor for the general experience of loneliness in all groups. We assumed that the presence of support from friends helps reduce the level of feeling lonely, regardless of the pandemic factor. The revealed tendency is consistent with the data of other researchers, which show a significant role of social support in reducing the level of loneliness [35].

Our study has several limitations. Firstly, we used cross-sectional data, and thus we cannot declare any dynamics or changes of the characteristics of loneliness. We can only discuss the between-sample differences. Another limitation is related to the quality of relationships that were associated with loneliness: family, friends, and romantic relationships. Assessment of quality of these relationships appears to be a complicated problem, since researchers would need a universal instrument that would fit all types of relationships. We see this direction as a future line of research.

## 5. Conclusions

Loneliness is considered to be one of the biggest challenges of modern society. Its negative connotations, such as associations with depression and anxiety, are well established. At the same time, the widely used three-item UCLA scale hardly addresses the complexity of the phenomenon. Our results clearly demonstrated that people having similar mean scores of general loneliness can feel lonely with family but satisfied and fulfilled with close friends or vice versa.

Our study addressed three main questions: if the pandemic situation affected participants in our sample, if there were age effects in pandemic groups and if the general experience of loneliness variance was explained by the same predictors in all defined groups. We concluded that pandemic effects were rather neutral or positive for our participants. Consistent with Sutin [17], we argue that the dramatic health threat caused by COVID-19 led to closer relations within families, more objective time spent together, and more concern for close relations. Analysis of age effects for mean levels of loneliness did not reveal any significant differences. Thus, consistent with Mund [8], we suggest that loneliness could be more related to life experiences than to age. The pandemic could be one such experience illuminating this causality. Finally, regression analyses revealed that predictors, explaining the variance of general experience of loneliness, were sensitive to pandemic situation and age. We assumed that, due to complex nature of the phenomenon, it can adjust to actual situation, and therefore, particular facets of loneliness can gain different a power depending on the current situation.

## Figures and Tables

**Table 1:** Demographic data for age groups in Study 1 and Study 2.

	Group 35–44				Group 45–59			
	*n*	M	SD	Males	*n*	M	SD	Males
Study 1	146	39.18	2.76	39.72	134	50.98	4.39	18.7
Study 2	72	39.78	2.54	45.83	105	51.17	4.22	43.8

*n*—number of participants in the group, M—mean age, SD—standard deviation for mean age, Males—percentage of males in the group.

**Table 2:** Descriptive statistics and values of *t*-test for loneliness characteristics in pre-pandemic and pandemic age groups.

Loneliness Characteristics	Age Groups	Pre-Pandemic		Pandemic		*t*	*p*	Bootstrap CI (95%)	
		M	SD	M	SD			Low Limit	High Limit
Family emotional loneliness	35–44	11.28	4.82	9.63	3.84	2.345	0.019	0.109	2.062
	45–59	11.75	5.53	10.89	5.31				
Non-family emotional loneliness	35–44	11.19	5.66	10.71	4.02	0.586	0.558	−0.545	1.194
	45–59	11.28	5.29	11.11	4.43				
Loneliness in romantic relationships	35–44	10.34	4.20	7.49	4.35	7.028	0.000	1.990	3.479
	45–59	10.01	4.19	7.41	4.38				
Romantic emotional loneliness	35–44	8.57	4.27	7.25	4.16	1.627	0.104	−0.119	1.338
	45–59	8.59	3.72	8.44	3.69				
General experience of loneliness	35–44	12.25	4.41	10.89	3.87	2.402	0.017	0.198	1.781
	45–59	12.70	4.93	11.86	3.61				
Dependence on communication	35–44	13.78	5.33	13.07	4.75	−0.152	0.879	−0.993	0.800
	45–59	13.08	4.94	13.83	4.27				
Positive loneliness	35–44	25.83	6.17	24.35	6.50	2.870	0.005	0.520	2.767
	45–59	25.83	5.94	24.07	5.98				

Note. Multivariate ANOVA (MANOVA) did not show any significant differences for factors “Age” and “Pandemic period × Age”. M—mean score, SD—standard deviation, *p*—*p*-value, *t*—*t*-value.

**Table 3:** Regression analysis of loneliness variables in age groups in pre-pandemic sample.

Loneliness Characteristics	B	SE	β	*p*	Bootstrap CI (95%)		R^2^
					Low Limit	High Limit	
Age Group 35–44							
Dependence on communication	0.277	0.056	0.335	0.000	0.158	0.413	0.621
Positive loneliness	0.153	0.050	0.214	0.003	0.068	0.255	
Family emotional loneliness	0.346	0.052	0.377	0.000	0.207	0.470	
Non-family emotional loneliness	0.215	0.048	0.275	0.000	0.115	0.354	
Romantic emotional loneliness	0.241	0.075	0.234	0.002	0.059	0.409	
Age Group 45–59							
Family emotional loneliness	0.298	0.061	0.334	0.000	0.163	0.429	0.611
Non-family emotional loneliness	0.407	0.069	0.437	0.000	0.235	0.580	
Romantic emotional loneliness	0.196	0.085	0.148	0.024	0.050	0.359	

Note. Only statistically significant predictors were included in the table. B—Beta, SE—standard error, β—standardized Beta, *p*—exact statistics for significance level.

**Table 4:** Regression analysis of the loneliness variables in age groups in pandemic sample.

Loneliness Characteristics	B	SE	β	*p*	Bootstrap CI (95%)		R^2^
					Low Limit	High Limit	
Age Group 35–44							
Non-family emotional loneliness	0.494	0.103	0.514	0.000	0.235	0.698	0.523
Age Group 45–59							
Dependence on communication	0.136	0.063	0.161	0.032	0.013	0.263	0.563
Positive loneliness	0.099	0.046	0.164	0.034	0.000	0.195	
Family emotional loneliness	0.237	0.057	0.348	0.000	0.101	0.363	
Non-family emotional loneliness	0.284	0.061	0.348	0.000	0.119	0.456	

Note. Only statistically significant predictors are included in the table. B—Beta, SE—standard error, β—standardized Beta, *p*—exact statistics for significance level.

## Data Availability

The data presented in this study are available on request from the corresponding author.
